# Navigating anatomical challenges of minimally invasive mitral valve replacement in a patient with severe scoliosis

**DOI:** 10.1093/jscr/rjaf369

**Published:** 2025-06-05

**Authors:** Henri Bartolozzi, Edward Bartolozzi, Darragh Rice, Ronan J Kelly, Mattia Glauber

**Affiliations:** Department of Cardiothoracic Surgery, University Hospital Galway, Newcastle Rd, Galway, H91YR71, Ireland; Faculty of Medicine, Trakia University, Armeyska St 11, Stara Zagora, 6000, Bulgaria; Faculty of Medicine, Trakia University, Armeyska St 11, Stara Zagora, 6000, Bulgaria; Department of Cardiothoracic Surgery, University Hospital Galway, Newcastle Rd, Galway, H91YR71, Ireland; Department of Cardiothoracic Surgery, University Hospital Galway, Newcastle Rd, Galway, H91YR71, Ireland; Department of Cardiothoracic Surgery, University Hospital Galway, Newcastle Rd, Galway, H91YR71, Ireland

**Keywords:** minimally invasive, mitral valve replacement, severe scoliosis, mechanical valve, septal myectomy

## Abstract

Minimally invasive mitral surgery is increasingly preferred due to reduced trauma and faster recovery. However, its technical complexity is exacerbated in patients with anatomical anomalies like scoliosis, affecting up to 8.3% of the elderly population. Severe scoliosis causes significant thoracic distortion and alters the positioning of cardiovascular structures, complicating access, visualization, and instrumentation. These factors make minimally invasive mitral valve replacement more complex, as this approach relies on optimal exposure of the mitral valve through a limited incision. We present the case of a 71-year-old female with severe scoliosis undergoing minimally invasive mitral valve replacement with a mechanical prosthesis and septal myectomy. Access was achieved via a 5 cm right mini-thoracotomy in the second intercostal space. This case report discusses the surgical challenges encountered and the unique considerations and adjustments required in performing cardiac surgery in patients with altered thoracic anatomy, and the importance of preoperative assessment and intraoperative flexibility.

## Introduction

Scoliosis, a condition affecting ⁓0.8% of the general population, significantly distorts normal thoracic and cardiac anatomy. It is commonly defined by a Cobb angle ˃10°, while severe scoliosis is classified by a Cobb angle of 45°–60° [1]. Minimally invasive mitral valve surgery has gained traction due to its advantages, including reduced surgical trauma, shorter hospital stays, and faster recovery. However, these benefits are counterbalanced by the technical complexities of the procedure, particularly when anatomical anomalies like scoliosis are present. In elderly populations, scoliosis has a prevalence of up to 8.3% [2]. These spinal deformities distort thoracic anatomy and cardiac positioning, significantly complicating the approach to minimally invasive mitral valve replacement (MVR).

In such patients, this leads to restricted access, impaired visualization, and altered positioning of the cardiovascular structures. Moreover, achieving precise mechanical valve alignment can be extremely challenging, requiring advanced surgical techniques, detailed preoperative imaging, and highly customized planning.

## Case presentation

**Figure 1:**
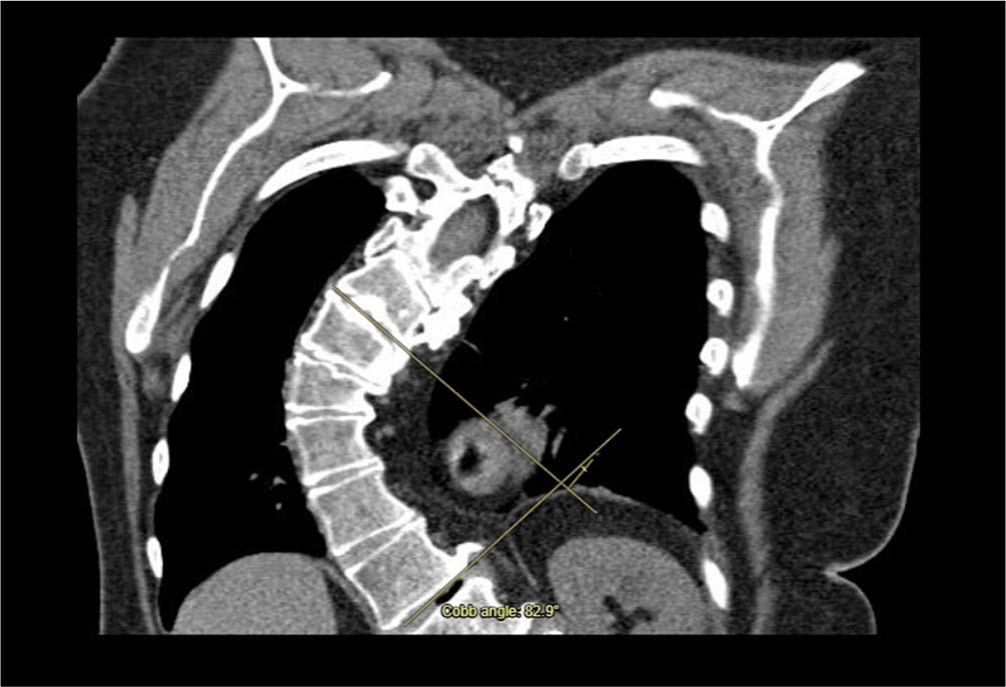
Coronal computed tomography (CT) with measurement of the cobb angle.

A 71-year-old female was referred to the heart team for elective minimally invasive MVR and septal myectomy. She presented with severe mitral regurgitation and hypertrophic obstructive cardiomyopathy (HOCM). Her body surface area was 1.51 m^2^, and BMI was 29.42. Critically, her scoliosis had a Cobb angle of 80° (Fig. 1), qualifying as extremely severe.

**Figure 2:**
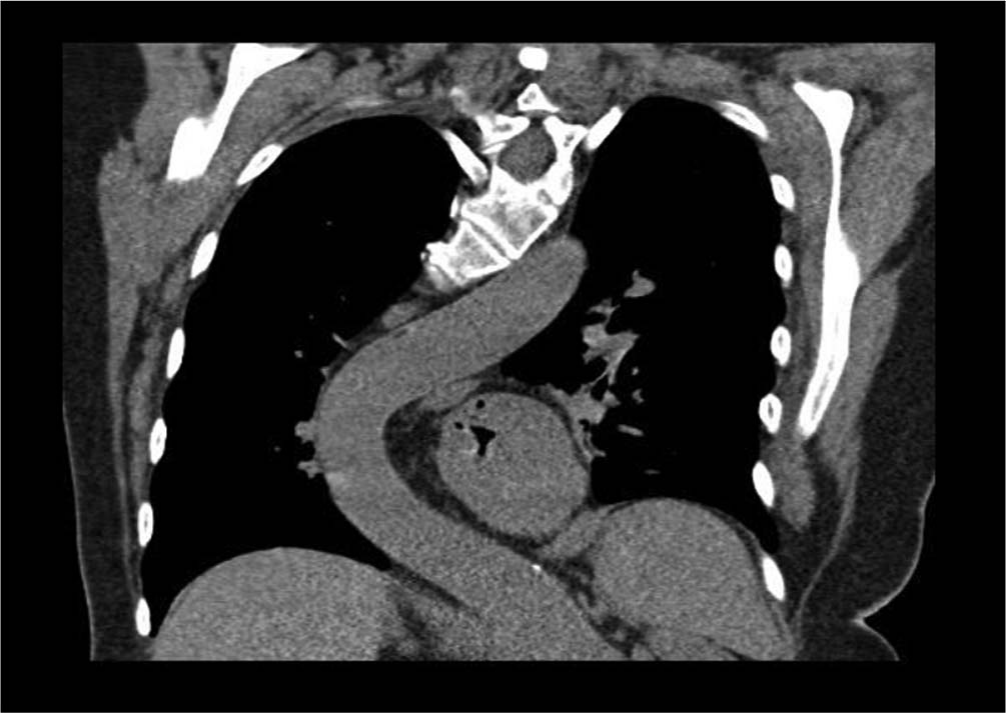
Coronal CT showing curvature of the thoracic aorta tortuosity compressing the distal esophagus and causing chronic left lower lobe segmental atelectasis.

The patient’s comorbidities were extensive and included: heart failure with preserved ejection fraction (HFpEF), atrial fibrillation, left atrial enlargement, Barrett’s esophagus, hypertension, hypercholesterolemia, multiple benign hepatic cysts, osteoporosis, a large sliding hiatal hernia containing the gastric fundus and celiac artery, and descending thoracic aortic tortuosity causing chronic left lower lobe segmental atelectasis (Fig. 2).

**Figure 3:**
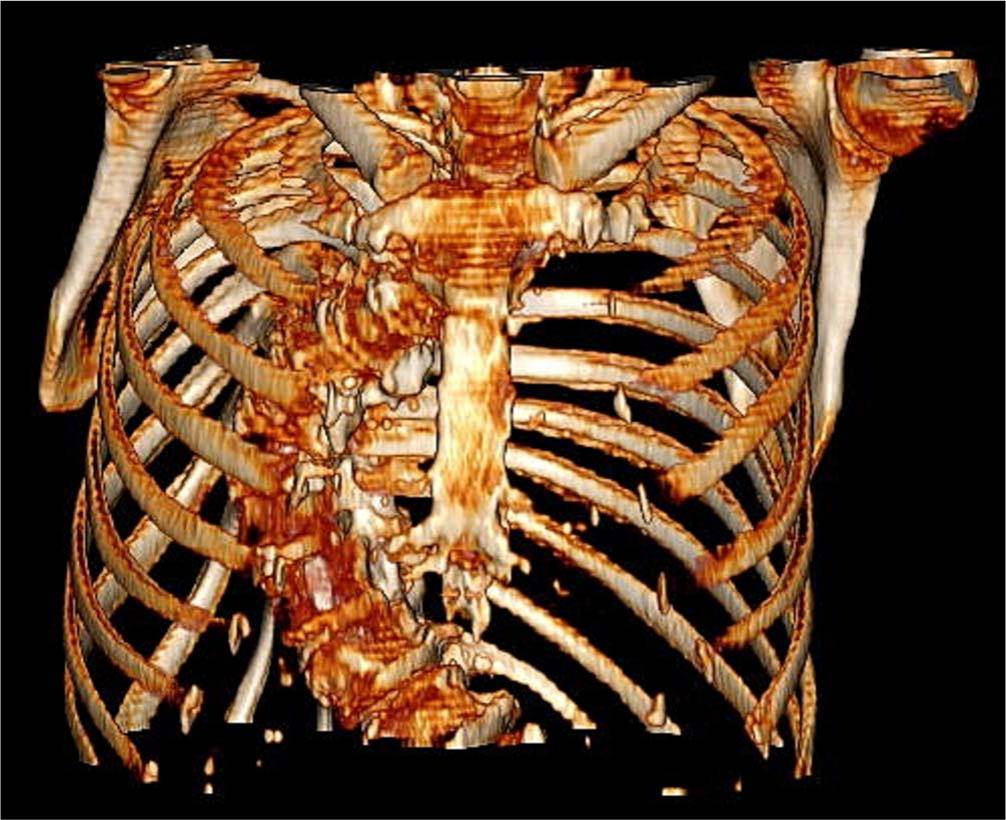
Pre-operative 3D reconstruction of patients thorax from CT.

**Figure 4:**
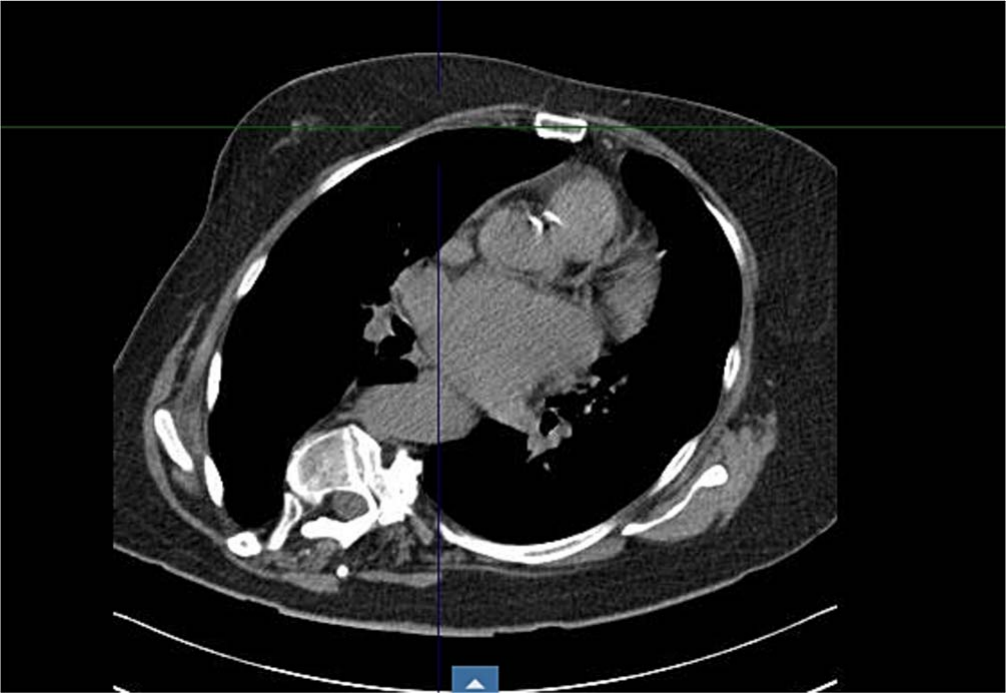
Axial CT at level of 2nd ICS.

Preoperative imaging highlighted this significant spinal deformity and its impact on cardiac orientation and accessibility (Fig. 3). The scoliosis increased the complexity of the surgery, necessitating customized operative strategies. The patient underwent minimally invasive MVR and septal myectomy through a right mini thoracotomy. A mechanical valve was selected as a redo would be near impossible due to the cases uniqueness and inherent difficulty. Operative technique involved a 5 cm skin incision on the right upper breast at the 2nd ICS for a working port (Fig. 4), with additional ports in the 2nd and 4th ICS for camera and CO₂ venting. Extracorporeal circulation was established via femoral–femoral bypass through a groin incision to access the left femoral vessels for cannulation.

Intraoperative findings included an overinflated right lung, left-rotated and enlarged heart, and a severely dilated atriua. There was evidence of pulmonary hypertension with dilated pulmonary arteries. The mitral valve displayed fibroelastic degenerative disease with very thin leaflets and multiple chordal ruptures on P2. The annulus, though not calcified, was notably dilated. LV function was mildly reduced, the left appendage was free of thrombi, and the septum was hypertrophic and fibrotic.

Once traction and exposure of left atrium and aorta were established, the cardioplegia needle was introduced into the aortic root and the aorta was cross-clamped using the Glauber clamp. A left atriotomy allowed mitral valve exposure. After evaluating the mechanism of disease, both leaflets were resected for a mechanical ON-X 25–33 prosthesis. Fixation was achieved using 17 U Ethibond sutures with pledgets fixed with Corknot device. The septal myectomy was performed via the mitral valve before implantation.

### Outcome and follow ups

Despite these challenges, the operation was completed without any major complications, and the patient experienced a smooth recovery. She was transferred to the Cardiothoracic Intensive Care Unit, then transferred to the cardiothoracic ward on the 2nd post-operative day where warfarin therapy was initiated. She recovered well, with no complications and was discharged on the 9th postoperative day once INR levels had been optimized.

At follow-up, postoperative echocardiography showed a max gradient of 11 mmHg, a mean gradient of 5.2 mmHg, mean velocity 1.03 m/s and a velocity-time integral of 30.08 cm, resulting in a satisfactory mitral valve replacement for such a complex patient.

## Discussion

Scoliosis, defined as a lateral spinal curvature exceeding 10°, affects between 0.5% and 3% of the general population, with prevalence influenced by age and severity. It is most commonly identified in adolescence, particularly among females, who have a higher risk for progression [3]. Among the elderly, scoliosis prevalence increases to an estimated 8.3%, often due to degenerative spinal changes [1].

Idiopathic scoliosis accounts for 80%–90% of cases, typically beginning in adolescence. Degenerative scoliosis primarily affects older adults as a result of intervertebral disc degeneration and facet joint arthritis [4]. In adults, comorbidities like osteoporosis and kyphosis frequently accompany scoliosis, further complicating treatment [5].

In patients with severe scoliosis, distorted thoracic anatomy and altered cardiac positioning present significant challenges for minimally invasive cardiac surgery, especially MVR. These anatomical complexities complicate access and visualization, necessitating a highly adaptable approach. While technically demanding, minimally invasive MVR offers advantages like reduced trauma and faster recovery, provided the surgical team uses flexible techniques and precise preoperative imaging to understand each patient's unique anatomy. Tailored strategies, such as limited incisions, specific cannulation routes, and flexible instrumentation, have proven to overcome scoliosis-related obstacles. This case underscores the importance of thorough planning and adaptability, suggesting that further research into minimally invasive approaches for scoliosis patients could improve outcomes in similar cases [6, 7].

### Tailored surgical approach

Access to the heart was achieved through a 5 cm mini thoracotomy on the right upper breast along the anterior axillary line at the level of the 2nd Intercostal space. Two ports were established in the 2nd and 4th ICS for a camera port and venting respectively along the middle axillary line. Extra corporal circulation was established via femoral—femoral access. Prior to valve implantation, a trans mitral septal myectomy was preformed due to the extensive HOCM. An ON-X 25–33 mechanical valve was fixed with 17 U Ethibond sutures with pladgets fixed with Corknot device.

## Conclusion

This case highlights that, with careful planning, customized surgical strategies, and adaptability, minimally invasive mitral valve replacement can be successfully performed in patients with severe thoracic deformities. It demonstrates how advances in cardiac surgery can accommodate anatomical challenges, delivering safe and effective outcomes for high-risk patients. It underscores the critical role of preoperative imaging and intraoperative flexibility in managing complex anatomical cases.
